# Content and quality of smartphone applications for bariatric surgery: A review and content analysis

**DOI:** 10.1016/j.pecinn.2025.100391

**Published:** 2025-04-08

**Authors:** Irma Hellbrecht, Nadja Könsgen, Alina Weise, Fabian Schlumberger, Dawid Pieper, Jessica Breuing

**Affiliations:** a Institute for Research in Operative Medicine (IFOM), Witten/Herdecke University, Germany; b Faculty of Health Sciences Brandenburg, Brandenburg Medical School (Theodor Fontane), Institute for Health Services and Health System Research, Rüdersdorf, Germany; c Center for Health Services Research, Brandenburg Medical School (Theodor Fontane), Rüdersdorf, Germany

**Keywords:** Bariatric surgery, Obesity, Digital applications, Patient information

## Abstract

**Objective:**

Our aim was to provide a descriptive analysis of the content and quality of bariatric apps available in Germany.

**Methods:**

From November 2022 to February 2023, apps available in German used in pre- or postoperative care were searched in the Google Play, the Apple App Store, and the Register for Digital Health Applications (DHA). One author reviewed titles and summary pages. Relevant apps were downloaded and two authors assessed their eligibility. Additionally, the authors independently screened records indexed in Medline/Embase. Besides a summary of the app content, a quality assessment was performed using two checklists (the Action Alliance for Patient Safety Checklist (APS); the Mobile App Rating Scale (MARS).

**Results:**

38 potentially relevant apps were identified, *n* = 3 were included. Functionality was good (MARS score 13–18/20). Content quality was variable (MARS score 7–19/35). Moreover, the apps' content lacked references and varied in scope.

**Conclusion:**

There are few apps in the field of BS available and the quality of their content is moderate to low. The evidence base remains unclear due to a lack of sources.

**Innovation:**

This is the first structured assessment of bariatric apps in Germany using validated checklists. The results provide a foundation for evidence-based, patient-centered app development in bariatric care and thus represent an important digital innovation in this field.

## Highlights

• Few German bariatric app identified through app store and database search.
• Functionality was good, content quality was variable.
• The apps' content lacked references and varied in scope.

## 1 Introduction

Obesity is a growing problem worldwide with various treatment options. Compared to conservative therapy, bariatric surgery (BS) is effective [1], but it is associated with side effects such as dietary changes [2,3], lifelong substitution of macro- and micronutrients [4], or psychosocial changes [5]. Accordingly, patients have a high information need [6]. Health information can support informed decision making and can help to improve health literacy, which is defined as “the degree to which individuals have the capacity to obtain, process, and understand basic health information and services needed to make appropriate health decisions” [7]. High health literacy is associated with improved weight loss after BS [8]. Accordingly, health information may also improve health-related outcomes in BS.

Reliable health information is most likely provided by healthcare professionals [9]. However, digital information sources are also used. Patients obtain information via different sources. Bariatric patients use web-based information [10], find motivation for weight loss through online forums [11] or monitor their nutritional intake with digital health applications (hereinafter called apps) [12]. Apps are digital medical devices that address diagnosis and management of diseases and are primarily used by patients [13]. Apps and web-based interventions are emerging as promising digital health tools to enhance perioperative care by promoting patient engagement, self-management, and improved health outcomes in other surgical setting [14].

However, information in apps or social media can also provide misinformation in the absence of a health care provider [15] or evidence to support it. As health care pathways and information provision in BS by healthcare providers appear to be heterogeneous in Germany [16], apps might be an additional information source for patients. High digital health literacy helps patients to process information, includes the ability to communicate digitally with lay users and health professionals and helps utilize the processed information [17]. Smartphone ownership is common in bariatric patients and apps are well accepted by dietitians [12], but to implement and adopt apps, they must be tailored to the user's needs [18]. Therefore, it is important to identify and eliminate barriers in information provision and app implementation**.** In the United Kingdom, there have been attempts to consider patients' needs when designing digital health technology in pre- and post-operative care [19].

There are some challenges in comparing apps internationally. One challenge is downloading a foreign app in Germany. There may be differences in the health care system between countries, resulting in differences in the delivery of information. Therefore, this review is limited to apps in the German context. To date, little is known about the number, availability, use, and content of bariatric apps in Germany. Therefore, the aim of this review is to conduct a descriptive analysis of their content and quality.

## 2 Methods

### 2.1 Study design

We conducted a descriptive analysis of the content and quality of apps for bariatric patients available in German. Two app stores were searched to identify relevant apps. We analyzed the app content and performed a quality assessment using the checklist for the use of apps by the German Coalition for Patient Safety (German: Aktionsbündnis Patientensicherheit APS) [20] and the Mobile App Rating Scale (MARS) [21]. We have published an a priori protocol [22] and followed PRISMA in the reporting of our study [23]. As this is not a systematic review evaluating a health care intervention, not all PRISMA items were applicable. This research did not receive any specific grant from funding agencies in the public, commercial, or not-for-profit sectors.

### 2.2 Eligibility criteria

Eligible apps had to address patients undergoing or interested in information about BS (pre- or postoperatively) and had to be available in German. Apps were included regardless of costs. Apps that did not specifically focus on BS (e.g., general dietary, fitness or health promoting apps) were excluded. We also excluded apps that were no longer available or inaccessible (e.g., doctor's prescription or login details necessary) or directed only at health care professionals.

### 2.3 Information sources and search strategy

We searched the German Google Play and Apple App stores between November 2022 and February 2023 using the following BS-related terms: bariatric, bariatric surgery, obesity, obesity surgery, weight loss surgery, gastric banding, gastric sleeve, gastric balloon and gastric bypass (searched in German). We also checked the suggested apps in the “similar apps” tab and screened the German Digital health applications (DHA) registry [13]. Secondly, we conducted a systematic literature search on 11th November 2022 using Medline (via Ovid) and Embase (via Elsevier) to identify further bariatric apps. The search strategy combined two search strings using relevant text- and keywords (see Appendix A.1).

### 2.4 Selection process

We screened the titles and overview pages of apps in the app stores and in the DHA registry. One reviewer (IH) checked the Google Play store and the DHA registry using an Android device and another reviewer (JB) screened the Apple app store using an Apple device. At this stage both reviewers discussed the relevance of identified apps. Apps considered relevant were downloaded and assessed for eligibility by both reviewers. We contacted app developers if apps were only accessible by prescription or login details. Contact details were obtained from app stores or by searching the internet.

Two reviewers independently performed the title and abstract (IH, JB) and full text screening (IH, FS) of the MEDLINE/Embase hits. Disagreements were resolved by discussion. If no consensus was achieved, a third author was involved. Apps identified through literature search had to be available in one of the aforementioned apps stores.

### 2.5 Data extraction

One reviewer (IH) extracted data from the included apps, the corresponding overview pages, and studies from the database search by using an a priori developed data extraction form. A second reviewer (JB, FS) verified data extraction. We extracted the following items: name and version of app, app store/platform, developer, date of release/update, app rating (number of ratings), available languages, and price. We categorized the apps according to the phase of care they referred to (pre-operative, post-operative, or both). In addition, we extracted the surgical procedures addressed by the apps and categorized whether the apps had the following functions: provision of educational information, calculation of body mass index (BMI), self-monitoring, reminder, frequently asked questions (FAQ), contact addresses, and digital patient record. Finally, we provided a short summary of their content.

### 2.6 Quality assessment

One reviewer (IH) performed quality assessment of the included apps with the MARS tool [21] and APS checklist [20] and a second reviewer (JB) verified quality assessment. We only applied section B “Functionality” (4 questions and a total score of max. 20) and section D “Information” (7 questions and a total score of max. 35) of the MARS tool, since the other domains were not relevant to our research question. We summed each item's score per domain and reported an overall score per domain. There was one item in the “information” domain assessing the visual information of apps. For apps including illustrations and videos, we calculated an individual score for illustration and for videos due to differences in the quality. We then calculated the overall quality score. The APS checklist consists of the following eight domains: purpose and functionality, quality and evaluation, ratings by other users, quality certificates and certification marks, data privacy notice, access to functionality and data, imprint, funding, and financial background. We entered our quality assessment for each app on the website, to determine whether the apps meet the quality requirements of the APS checklist.

## 3 Results

### 3.1 Selection

**Fig. 1:**
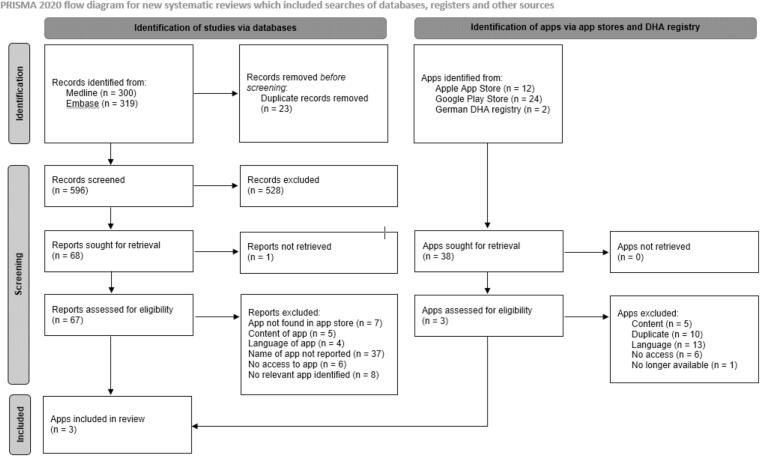
PRISMA flow chart (modified).

The search in the app stores yielded 38 potentially relevant apps. We downloaded these apps and contacted the developers of 9 apps. We only received feedback from five developers. They either described BS as a contraindication or confirmed that there was no possibility to access the app due to technical reasons. Additionally, we requested test versions of two apps, but only received access to one*.* After checking the test version, we excluded the app because its content was not tailored to bariatric but obese patients (no operation planed or required). In total, we excluded 35 apps after screening the app stores and the DHA registry. The reasons for exclusion are listed in Fig. 1 and Appendix A.2. The selection process is illustrated in Fig. 1. Finally, we included the following three apps (developer): *Agrundo (ClinicAdvisor von der Groeben)* [24]*, Adipositas-Behandlungen* [obesity therapy] *(Instituto de Obesidad)* [25] and *Adipositas-Chirurgie* [bariatric surgery] *(Izmir Obezite Cerrahi)* [26]. The app *Adipositas-Chirurgie* [bariatric surgery] [26] is no longer available in the Google Play store.

The systematic literature search of the electronic databases identified 619 references. After excluding 23 duplicates, we screened 596 titles and abstracts and retrieved 67 reports. We excluded all reports due to the reasons listed in Fig. 1 and Appendix A.3.

### 3.2 Characteristics of included apps

All apps were available for Android devices, but only one app was also available for iOS devices [24]. They were published between 2016 and 2019 and were last updated between 2017 and 2021. See Table 1 for more information on the general characteristics.

**Table 1:** General characteristics of apps.

Name of app	Adipositas-Behandlungen [obesity therapy]	Adipositas-Chirurgie [bariatric surgery**]**	Agrundo
App-Store	Android	Android	Android/Apple
Developer	Instituto de Obesidad	izmir Obezite Cerrahi	ClinicAdvisor von der Groeben
Release date	November 29th 2017	December 22th 2019	March 24th 2016
Date of last update	November 29th	July 9th 2021	May 26th 2021
App rating	/	/	/
Number of ratings	0	0	0
Languages	German (translated from Spanish)	German (translated from Turkish)	German
Price	free	free	free
Focus of App	Information provision	Information provision	Self-monitoring
Pre-, postoperative care or both	Preoperative care	Both	Both
App functions			
Calculation of BMI	✓	✓	✓
Self-monitoring			✓
Reminder (e.g. medication)			✓
FAQ		✓	
Contact addresses	✓		✓
Digital patient record			✓

All apps were free to download. User ratings were not available. The app *Adipositas-Behandlungen* [obesity therapy] [25] was mainly targeted at preoperative patients, while the apps *Adipositas-Chirurgie* [bariatric surgery] [26] and *Agrundo* [24] included both preoperative and postoperative content. Two apps mainly focused on information provision [25,26], whereas the third app aimed at self-monitoring [24]. All apps contained educational information and enabled the calculation of the BMI. Contact details (i.e., postal address) were available for two apps [24,25] and a collection of FAQ was offered by one app [26]. The app *Agrundo* [24] enabled patients to self-monitor, provided reminders (e.g., to take medication) and allowed users to document their data (e.g. height, weight, comorbidities, surgical procedure) in a digital patient record. This app was more comprehensive compared to the other two apps that have been presumably translated automatically [25,26].

The app *Adipositas-Behandlungen* [obesity therapy] contains information on 12 different surgical procedures. The app provides explanatory text, photos and videos for each procedure and allows to calculate the BMI. Images lack captions and videos are mostly in Spanish or without sound.

The app *Aditositas-Chirurgie* [bariatric surgery] refers to general BS and contains behavioral recommendations and recommendations on nutrition before/after surgery. Users can take notes, use the FAQ section and keep a to-do list.

**Fig. 2:**
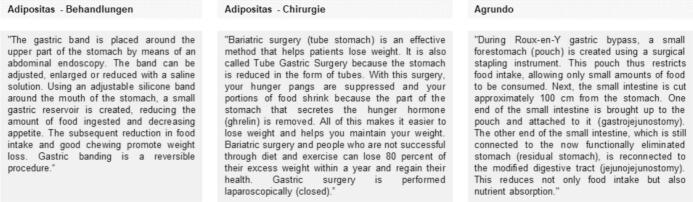
Examples of the apps' content. – Quotes translated with DeepL (https://www.deepl.com/de/translator).

The app *Agrundo* provides brief text, images and videos for gastric bypass and sleeve gastrectomy. Images do not contain explanations. Videos contain sound and explain the surgical procedures in an illustrative manner. Moreover, users can document their personal data, monitor health changes and receive reminders (push messages) to take medication. See Table 2 and Fig. 2 for a detailed description of the apps' content.

**Table 2:** Summary of the content of apps.

Name of app	Surgical procedures	Summary of Information/functionalities
Adipositas-Behandlungen [obesity therapy]	- Apollo endosleeve - Biliopancreatic diversion - Endobarrier - Endoluminal surgery (POSE) - External bypass (Aspire) - Gastric balloon (6 or 12 months) - Gastric balloon elipse - Gastric banding - Gastric bypass - Mini-gastric bypass - Gastric balloon (OBALON) - Sleeve gastrectomy	- Very brief information on surgical procedures: app points out whether surgery (yes/no), endoscopy (yes/no) or general anaesthesia (yes/no) are required for the surgical procedures - Patients can calculate their own BMI and compare it with a BMI suitable for the surgical procedure - Brief explanatory text, images and videos - Images do not contain explanations; videos are mostly in Spanish or without sound - Videos include schematic diagrams and explanations of an expert (in Spanish) - Link to the clinic's website and contact address in Spain (phone number, e-mail, postal address)
Adipositas-Chirurgie [bariatric surgery]	- BS in general - Sleeve gastrectomy	- Very brief information on BS - Behavioral recommendations and recommendations on nutrition before and after surgery - FAQ - No images or videos available - Patients can calculate their BMI - Options to take notes, To-do list and phone number (Turkish) - Presumable automatically translated information, partly incorrect regarding grammar and therefore difficult to understand
Agrundo	- Gastric bypass - Sleeve gastrectomy	- Information on gastric bypass surgery and sleeve gastrectomy - Text, images and videos - Images do not contain explanations; videos contain sound and explain the surgical procedures in an illustrative manner - Patients can document the following personal data: clinic, surgery date, surgeon, surgery method and comorbidities - Monitoring/documentation of BMI, body weight, diseases before/after surgery and medication plan - Regular reminders to take medication - Phone number and address to contact one clinic in Basel (Switzerland)

Overall, the apps provided brief information about BS. None of the apps offered any form of chat, either with other users, with healthcare professionals, or with presumably artificial intelligence.

### 3.3 Quality assessment of included apps

According to the MARS score, the overall rating of the domain “functionality” of the included apps was moderate to good. The features and components of all apps worked accurately and fast. However, there was no menu in the app *Adipositas-Chirurgie* [bariatric surgery], its structure was not intuitive and the FAQ section had a different design compared to the rest of the app. The app *Adipositas-Behandlungen* [obesity therapy] had no menu either, but the front page of the app was more intuitive and easier to use. Only *Agrundo* had a menu to switch between screens and components. The app was mostly consistent, although the designations of components were misleading in some cases (e.g., the data privacy note was located under the imprint tab). The overall quality of the domain “information” of the MARS tool was rather low. There were big differences in how the apps were described in the app stores. No description was available for the app *Adipositas-Chirurgie* [bariatric surgery]. The *Adipositas-Behandlungen* [obesity therapy] app lacked some information, although mentioned in the app description in the app store. However, *Agrundo* had a very accurate description in the app store. Due to the low quality of the translation of both apps *Adipositas-Behandlungen* [obesity therapy] and *Adipositas-Chirurgie* [bariatric surgery]*,* misunderstandings of the content may occur. *Agrundo* had a rather high quality of information, but only two surgical procedures were presented. The information of all apps was either very brief or appeared overwhelming in its presentation to the reviewers. Visual information was not always clear, since descriptions and explanations were missing for illustrations.

None of the apps met the quality requirements of the APS. There were no statements about limitations of apps and the last update was more than 6 months ago. Neither user ratings in the app stores, nor quality certificates were available. Only *Agrundo* provided a data privacy notice that was easy to find and included information about the data collection. No app contained information about the legal form of the provider and its financial background. However, the apps were neutral and there were no indications of commercial influencing. For details on the quality assessment of the included apps, see Appendix A.4 and A.5.

In addition to the aforementioned quality assessment, we noticed that none of the apps provided references for their content.

## 4 Discussion and conclusion

### 4.1 Discussion

We found only three apps relevant to patients with BS. Two seem to be translated automatically and were not developed for the German market/healthcare system. Therefore, the actual benefit for German patients is uncertain. The quality of the apps was moderate to low, depending on the domain. The apps vary in the amount of information provided, and none includes supporting references. This makes it difficult for users to verify the quality and authenticity of the content. In summary, no app in the German Apple and Google Play stores currently provides evidence-based patient information for the pre- and postoperative care of patients with BS. However, providing evidence-based patient information would serve the information need in Germany [6]. In 2020, the patient version of the German clinical guideline “Surgery of Obesity and Metabolic Diseases” was published, providing evidence-based information for patients undergoing BS [27].

Although online forums [11,28] and healthcare professionals are important sources of information [9], none of the included apps provided a tool for interacting with other patients or healthcare professionals, asking questions or sharing experiences. Future apps should address this.

The question is why there are so few suitable German apps for BS. During our research period, one of the apps (*Mein Magenbypass* [my gastric bypass]) was removed from the app store due to insufficient utilization. The developers stated three reasons for this: a low willingness to pay for the app, the small size of the German-speaking market and users' expectations for different content, like a weight-loss app. This might explain the high proportion of weight loss apps in both app stores. With two out of three apps only available in the Google Play Store, access to apps was also unequal. Barriers to accessing the apps must be eliminated, such as the app's availability in all major app stores.

### 4.2 Limitations

There were some technical barriers to researching the apps. Since we intended to check both the Apple and Google Play App Stores, we needed an appropriate mobile phone for each store (Apple/Android). App-Store screening was not performed independently because of limited resources due to lack of funding and because the smartphones used for app store screening were the researchers' personal devices.

Partially, the order of apps in the app stores changed within hours. There were no page numbers for search results, no automatic counting of results, and many obviously irrelevant results. This may not impact our results, but it led to increased screening challenges compared to screening within structured databases. Search results could not be exported, but had to be extracted manually. After the screening, we discovered the web-based AI “BrowseAI” (https://www.browse.ai/), which could be a useful tool for future app screening and data extraction, since the tool extracts content from any websites, such as Google Play Store, which has a browser-based version. However, Apple App Store screening is smartphone based, so this tool could not be used there.

The accessibility of some apps was limited because, as a registered German digital health application [13], they only grant prescription access. Even by contacting the provider, we were unable to gain access. Therefore, they are not included in this review. However, we assumed that the apps' content would address obesity treatment in general rather than BS, based on the summaries available on the app stores.

During the systematic literature search we excluded studies because the names of apps were not reported. However, we did not contact study authors, because there were limited resources due to missing funding. However, the systematic literature search had the advantage of allowing us to identify at an early stage apps in development that were not yet available in any of the app stores.

We have only searched for apps in German. Therefore, our results may not be fully applicable to non-German speaking countries. For example, two of the included apps seem to be translated automatically and therefore lacked in language quality.

The quality assessment was not independently due to limited resources, but the second reviewer verified the ratings of the first reviewer. Consensus was sought in case of any discrepancy.

### 4.3 Innovation

There is an ongoing study in Germany to implement a structured postoperative care system for BS, including an app for patients [29]. The study results have not yet been published, and despite contacting the research team, it was not possible to access the app. The results of this current study emphasize the requirement for a suitable app that is tailored to the specific needs of BS patients.

### 4.4 Conclusion

Apps can be an effective way to offer health-related patient information. However, only a few bariatric apps are available in German. They have a moderate quality and a restricted accessibility depending on the device system. The benefits of digitizing evidence-based information for patients, such as creating a patient-version app, might increase accessibility and individualization, while also reducing known barriers. This study's findings reinforce the need for a high quality, evidence-based app that addresses the specific needs of BS patients and is available in both app stores.

## Credit authorship contribution statement

**Irma Hellbrecht:** Writing – review & editing, Writing – original draft, Formal analysis. **Nadja Könsgen:** Writing – review & editing. **Alina Weise:** Writing – review & editing, Data curation. **Fabian Schlumberger:** Writing – review & editing, Formal analysis. **Dawid Pieper:** Writing – review & editing. **Jessica Breuing:** Writing – review & editing, Writing – original draft, Supervision, Methodology, Investigation, Formal analysis, Conceptualization.

## Declaration of competing interest

The authors declare that they have no known competing financial interests or personal relationships that could have appeared to influence the work reported in this paper.

## Appendix A Supplementary data

Supplementary material 1 Image 1

Supplementary material 2 Image 2

Supplementary material 3 Image 3

Supplementary material 4 Image 4

Supplementary material 5 Image 5
