# Supplementary material for: Content and quality of smartphone applications for bariatric surgery: A review and content analysis
Source: PEC Innov. 2025 Apr 8;6:100391. doi: 10.1016/j.pecinn.2025.100391 (PMC12023771; doi:10.1016/j.pecinn.2025.100391)
Supplement: Supplementary file 1 — Supplementary material 1 [file mmc1.docx]

**Appendix A.1** – Search strategy for studies on bariatric apps

Bariatric surgery and the other search string was based on a validated health app search filter for health apps [1].

MEDLINE (via Ovid)

Search date: 11.11.2022

| **Search** | **Query** | **Items** |
| --- | --- | --- |
| 1 | exp Bariatric Surgery/ | 32151 |
| 2 | exp Obesity/su [Surgery] | 24669 |
| 3 | ((bariatric or weight loss or weightloss or obesity or obese or anti obesity or antiobesity or restrictive or metabolic) adj4 surg*).ti,ab,kw. | 30050 |
| 4 | bariatric patient?.ti,ab,kw. | 1327 |
| 5 | (sleeve gastrectom* or gastric sleeve* or gastric balloon or roux-y or roux-en-y or omega-loop* or gastroplast* or gastric band* or duodenal switch* or gastric bypass or jejunoileal bypass or biliopancreatic bypass or biliopancreatic diversion* or gastrointestinal diversion*).ti,ab,kw. | 28580 |
| 6 | exp Biliopancreatic Diversion/ | 1092 |
| 7 | or/1-6 | 56289 |
| 8 | mobile applications/ | 10697 |
| 9 | exp Internet/ | 94635 |
| 10 | exp cell phone/ | 21263 |
| 11 | exp computers, handheld/ | 12238 |
| 12 | medical informatics applications/ | 2550 |
| 13 | therapy, computer-assisted/ | 6966 |
| 14 | (app or apps).ti,ab. | 39527 |
| 15 | (online or web or internet or digital*).ti. | 127915 |
| 16 | ((online or web or internet or digital*) adj3 (based or application* or intervention* or program* or therap*)).ab. | 72295 |
| 17 | (phone* or telephone* or smartphone* or cellphone* or smartwatch*).ti. | 25377 |
| 18 | ((phone* or telephone* or smartphone* or cellphone* or smartwatch*) adj3 (based or application* or intervention* or program* or therap*)).ab. | 15473 |
| 19 | (mobile health or mhealth or m-health or ehealth or e-health or emental or e-mental).ti. | 7727 |
| 20 | ((mobile health or mhealth or m-health or ehealth or e-health or emental or e-mental) adj3 (based or application* or intervention* or program* or therap*)).ab. | 5232 |
| 21 | (mobile* adj3 (based or application* or intervention* or device* or technolog*)).ti,ab. | 19735 |
| 22 | or/8-21 | 320307 |
| 23 | 7 and 22 | 300 |

Embase via Elsevier

Search date: 11.11.2022

| **Search** | **Query** | **Items** |
| --- | --- | --- |
| 1 | 'bariatric surgery'/exp | 53861 |
| 2 | 'obesity'/dm_su | 12491 |
| 3 | 'gastric bypass surgery'/exp | 30194 |
| 4 | 'jejunoileal bypass'/exp | 750 |
| 5 | ((bariatric OR 'weight loss' OR weightloss OR obesity OR obese OR 'anti obesity' OR antiobesity OR restrictive OR metabolic) NEAR/4 surg*):ti,ab,kw | 54388 |
| 6 | 'bariatric patient$':ti,ab,kw | 2836 |
| 7 | 'sleeve gastrectom*':ti,ab,kw OR 'gastric sleeve*':ti,ab,kw OR 'gastric balloon':ti,ab,kw OR 'roux-y':ti,ab,kw OR 'roux-en-y':ti,ab,kw OR 'omega-loop*':ti,ab,kw OR gastroplast*:ti,ab,kw OR 'gastric band*':ti,ab,kw OR 'duodenal switch*':ti,ab,kw OR 'gastric bypass':ti,ab,kw OR 'jejunoileal bypass':ti,ab,kw OR 'bilippancreatoc bypass':ti,ab,kw OR 'biliopancreatic diversion*':ti,ab,kw OR 'gastrointestinal diversion*':ti,ab,kw | 51061 |
| 8 | #1 OR #2 OR #3 OR #4 OR #5 OR #6 OR #7 | 91024 |
| 9 | 'mobile application'/exp | 22119 |
| 10 | 'internet':de | 127602 |
| 11 | 'mobile phone'/exp | 42620 |
| 12 | 'text messaging':de | 7060 |
| 13 | 'personal digital assistant':de | 1772 |
| 14 | app:ti,ab OR apps:ti,ab | 56161 |
| 15 | online:ti OR web:ti OR internet:ti OR digital*:ti | 159962 |
| 16 | ((online OR web OR internet OR digital*) NEAR/3 (based OR application* OR intervention* OR program* OR therap*)):ab | 98881 |
| 17 | phone*:ti OR telephone*:ti OR smartphone*:ti OR cellphone*:ti OR smartwatch*:ti | 30807 |
| 18 | ((phone* OR telephone* OR smartphone* OR cellphone* OR smartwatch*) NEAR/3 (based OR application* OR intervention* OR program* OR therap*)):ab | 20723 |
| 19 | 'mobile health':ti OR mhealth:ti OR 'm-health':ti OR ehalth:ti OR 'e-health':ti OR emental:ti OR 'e-mental':ti | 6484 |
| 20 | (('mobile health' OR mhealth OR 'm-health' OR ehealth OR 'e-health' OR emental OR 'e-mental') NEAR/3 (based OR application* OR intervention* OR program* OR therap*)):ab | 5876 |
| 21 | (mobile* NEAR/3 (based OR application* OR intervention* OR device* OR technolog*)):ti,ab | 24350 |
| 22 | #9 OR #10 OR #11 OR #12 OR #13 OR #14 OR #15 OR #16 OR #17 OR #18 OR #19 OR #20 OR #21 | 424913 |
| 23 | #8 AND #22 | 619 |
| 24 | #23 NOT [medline]/lim | 319 |

1. Ayiku, L., et al., *The NICE MEDLINE and Embase (Ovid) health apps search filters: development of validated filters to retrieve evidence about health apps.* Int J Technol Assess Health Care, 2020. **37**: p. e16.
