# Supplementary material for: Content and quality of smartphone applications for bariatric surgery: A review and content analysis
Source: PEC Innov. 2025 Apr 8;6:100391. doi: 10.1016/j.pecinn.2025.100391 (PMC12023771; doi:10.1016/j.pecinn.2025.100391)
Supplement: Supplementary file 2 — Supplementary material 2 [file mmc2.docx]

**Appendix A.2 – Screening of bariatric apps in the App Stores**

| Name of app | App developer | Publication date of app [dd.mm.yyyy] | German search term | Date of search [dd.mm.yyyy] | Inclusion after app store screening [y/n] | Inclusion after app download [y/n/Inquiry*] | Final Inclusion [y/n] | Reason for exclusion |
| --- | --- | --- | --- | --- | --- | --- | --- | --- |
| Apple App Store | | | | | | | | |
| Zanadio | aidhere GmbH | Vor 3 Monaten | Adipositas | 17.11.2022 | y | Inquiry | n | Content |
| Oviva Direkt | Oviva AG | Vor 2 Tagen | Adipositas | 17.11.2022 | y | Inquiry | n | No access |
| Clinical PracticeGuideline | The Endocine Society | NR | Bariatrisch | 21.12.2022 | y | n | n | Language |
| Bariatric Times | Matrix Medical Communication | NR | Bariatrisch | 21.12.2022 | y | n | n | Language |
| Bariatric World | Honeycommb | NR | Bariatrisch | 21.12.2022 | y | n | n | Language |
| Planet Bariatrics | planetbariatrics | NR | Bariatrisch | 21.12.2022 | y | n | n | Language |
| Ultimate Bariatrics | Virtual Health Partners Inc | NR | Bariatrisch | 21.12.2022 | y | Inquiry | n | No access |
| SBCBM 2019 | Sociedade Brasileira de Chirurgia Bariatrica e Metabolica | NR | Bariatrisch | 21.12.2022 | y | n | n | Language |
| Bariatric Meal Timer | Webb Dsgn | NR | Bariatrisch | 21.12.2022 | y | n | n | Language |
| Agrundo | ClinicAdvisor von der Groeben | NR | Bariatrisch | 21.12.2022 | y | y | y | / |
| Bariatric Eating | BariatricPaL LLC | NR | Bariatrisch | 21.12.2022 | y | n | n | Language |
| My Weight Loss Journey | The University of Michigan | NR | Bariatrisch | 21.12.2022 | y | n | n | Language |
| Google Play Store | | | | | | | | |
| Adipositas-Behandlungen | Instituto de Obesidad | 29.11.2017 | Adipositas | 29.11.2022 | y | y | y | / |
| Adipositas-Chirurgie | Izmir Obezite Cerrahi | 22.12.2019 | Adipositas | 29.11.2022 | y | y | y | / |
| Zanadio | aidhere GmbH | 15.10.2020 | Adipositas | 29.11.2022 | y | n | n | Duplicate |
| Noom: Gewicht & Health | Noom Inc. | 17.03.2011 | Adipositas | 29.11.2022 | y | n | n | Content |
| Oviva Direkt | Oviva AG | 04.10.2021 | Adipositas | 29.11.2022 | y | n | n | Duplicate |
| Oviva | Oviva AG | 15.08.2014 | Adipositas | 29.11.2022 | y | Inquiry | n | Content |
| BariBuddy | Baricol Bariatrics AB | 31.05.2018 | Adipositas | 01.12.2022 | y | n | n | Language |
| ACHT-Nachsorge | symeda GmbH | 28.01.2020 | Adipositas | 01.12.2022 | y | Inquiry | n | No access |
| BariatricPal | BariatricPal.com | 30.09.2013 | Adipositas | 01.12.2022 | y | n | n | Language |
| Bariatric IQ | NordClinic | 15.12.2015 | Adipositas | 01.12.2022 | y | n | n | Language |
| Deutsches GesundheitsPortal | HealthCom GmbH | 06.02.2018 | Adipositas | 01.12.2022 | y | n | n | Content** |
| Agrundo | ClinicAdvisor von der Groeben | 24.03.2106 | Adipositas | 01.12.2022 | y | n | n | Duplicate |
| Bariatric World | Honeycommb | 19.04.2022 | Bariatrisch | 08.12.2022 | y | n | n | Duplicate |
| Dr Bariatric | Jason Kuklinski | 26.01.2021 | Bariatrisch | 08.12.2022 | y | n | n | Language |
| Bariatric Meal Timer | Webb Dsgn | 26.02.2018 | Bariatrisch | 08.12.2022 | y | n | n | Duplicate |
| Bariatric Times | Matrix Medical Communication | 25.05.2018 | Bariatrisch | 08.12.2022 | y | n | n | Duplicate |
| Barilife | SBCBM | 12.05.2019 | Bariatrisch | 08.12.2022 | y | n | n | Language |
| Bariatric Keto | Bariatric Keto | 27.06.2018 | Bariatrisch | 08.12.2022 | y | Inquiry | n | No access |
| Bariatric Risk Assessment Tool | Proactiff Digital Healthcare Services | 21.03.2020 | Bariatrisch | 08.12.2022 | y | Inquiry | n | No access |
| Planet Bariatrics | planetbariatrics | 31.08.2021 | Bariatrisch | 08.12.2022 | y | n | n | Duplicate |
| BariFit | TRAINERIZE | 29.10.2016 | Bariatrisch | 08.12.2022 | y | Inquiry | n | No access |
| My Weight Loss Journey | The University of Michigan | 23.10.2020 | Bariatrisch | 08.12.2022 | y | n | n | Duplicate |
| EWLCA | CoachCare | 13.04.2020 | Magenballon | 09.12.2022 | y | n | n | Content |
| Mein Magenbypass | Praxis Verena Jaggi | 10.10.2015 | Magenbypass | 09.12.2022 | y | Inquiry | n | No longer available |

*Inquiry: App developer were contacted if potentially relevant apps were only accessible via prescription or login data.

**The app summarises the content of studies on various topics in plain language (including bariatric surgery), but does not represent standard care.

**Appendix – Screening of bariatric apps in the DHA registry**

| Name of DHA | App developer | First entry into the DHA-registry [dd.mm.yyyy] | Duration of entry into the DHA-registry | Inclusion after screening the registry [y/n] | Inclusion after app download [y/n] | Final Inclusion [y/n] | Reason for exclusion |
| --- | --- | --- | --- | --- | --- | --- | --- |
| Oviva Direkt für Adipositas | Oviva AG | 03.10.2021 | Permanently | y | n | n | Duplicate |
| Zanadio | aidhere GmbH | 22.10.2020 | Permanently | y | n | n | Duplicate |
