# Supplementary material for: Content and quality of smartphone applications for bariatric surgery: A review and content analysis
Source: PEC Innov. 2025 Apr 8;6:100391. doi: 10.1016/j.pecinn.2025.100391 (PMC12023771; doi:10.1016/j.pecinn.2025.100391)
Supplement: Supplementary file 3 — Supplementary material 3 [file mmc3.docx]

**Appendix A.3 – Excluded studies after database search**

| **Study** | **Reason for exclusion** |
| --- | --- |
| Ahsan SD, Sanyang N, Tsai AYC, Ramar S. Evaluation of patient centred digital information in bariatric surgery-a content analysis of mobile applications and web-based information. Obesity Surgery. 2021;31(SUPPL 1). | Name of app not reported |
| Al-Ozairi E, AlAwadhi MM, Al Kandari J, Taghadom E, Abdullah M, Le Roux CW. Photo-Assisted Dietary Method Improves Estimates of Dietary Intake Among People with Sleeve Gastrectomy. Obesity Surgery. 2019;29(5). | No relevant app identified |
| Aleman R, Matute CM, Mora MF, Frieder JS, Lo Menzo E, Szomstein S, et al. Patient therapeutic education: smartphone-based applications for the bariatric surgery patient. Surgical Endoscopy. 2019;33. | Name of app not reported |
| Aminian A, Clemence S, Alberts J, Schauer P, Brethauer S. Bariatric surgery decision-making calculator: A novel mobile app for evidence-based clinical practice. Surgery for Obesity and Related Diseases. 2017;13(10). | App not found in app store |
| Ash J, Gokani S, Kerry G, Zargaran A, Rasasingam D, Mittal A, et al. Assessing the educational requirements of bariatric surgery patients technology and bariatric surgery. Obesity Surgery. 2017;27(1). | Name of app not reported |
| Ashurst EJ, Jones RB. Is the Health App Challenge approach of patient-led application conception, development, and review worthwhile? Health Policy and Technology. 2017;6(1). | Name of app not reported |
| Bonn SE, Hult M, Spetz K, Lof M, Andersson E, Wiren M, et al. App Technology to Support Physical Activity and Intake of Vitamins and Minerals After Bariatric Surgery (the PromMera Study): Protocol of a Randomized Controlled Clinical Trial. JMIR Research Protocols. 2020;9(8). | App not found in app store |
| Connor K, Brady RR, Tulloh B, de Beaux A. Smartphone applications (apps) for bariatric surgery. Obesity Surgery. 2013;23(10). | No relevant app identified |
| Cossu L, Cappon G, Herzig D, Bally L, Facchinetti A. MULTI-MODAL MOBILE PLATFORM FOR THE INVESIGATION OF CLINICAL DISORDERS WITH GLYCAEMIC DISARRAYS. Diabetes Technology and Therapeutics. 2022;24(SUPPL 1). | App not found in app store |
| Deniz Dogan S, Arslan S. The Effects of e-Mobile Training and Consultancy Services on Bariatric Surgery Patients: A Randomized Clinical Trial. Obesity Surgery. 2022;32(11). | Name of app not reported |
| Dolan PT, Afaneh C, Dakin G, Pomp A, Yeo HL. Lessons Learned From Developing a Mobile App to Assist in Patient Recovery After Weight Loss Surgery. Journal of Surgical Research. 2019;244. | Name of app not reported |
| Elvin-Walsh L, Ferguson M, Collins PF. Nutritional monitoring of patients post-bariatric surgery: implications for smartphone applications. Journal of Human Nutrition & Dietetics. 2018;31(1). | Language of app |
| Estrade A, Montastier E, Turnin MC, Buisson JC, du Rieu MC, Tuyeras G, et al. An Application May Help Improve Protein Consumption after Bariatric Surgery. Obesity Surgery. 2019;29(6). | Name of app not reported |
| Gamme G, Kolozsvari N, Jarrar A, Neville A. Effect of Mobile Electronic Health Application on Postoperative Outcomes of Bariatric Surgery Patients. Surgery for Obesity and Related Diseases. 2019;15(10). | Language of app |
| Graham Y, Hayes C, Mahawar K, Small P, Attala A, Seymour K, et al. What do uk allied health practitioners think of the place of social media and technology for bariatric patient support? integrated health/multidisciplinary care. Obesity Surgery. 2017;27(1). | Name of app not reported |
| Graham YNH, Hayes C, Mahawar KK, Small PK, Attala A, Seymour K, et al. Ascertaining the Place of Social Media and Technology for Bariatric Patient Support: What Do Allied Health Practitioners Think? Obesity Surgery. 2017;27(7). | App not found in app store |
| Hayotte M, Iannelli A, Negre V, Pradier C, Therouanne P, Fuch A, et al. Effects of technology-based physical activity interventions for women after bariatric surgery: study protocol for a three-arm randomised controlled trial. BMJ Open. 2021;11(7). | Name of app not reported |
| Heuser J, Maeda A, Masino C, Jackson T, Okrainec A. Reducing readmission following bariatric surgery: Is there an app for this? Surgical Endoscopy. 2019;33. | Name of app not reported |
| Heuser J, Maeda A, Yang L, Masino C, Duggal S, Jackson T, et al. Impact of a Mobile App to Support Home Recovery of Patients Undergoing Bariatric Surgery. Journal of Surgical Research. 2021;261. | Language of app |
| Hult M, Bonn SE, Andersson E, Spetz K, Lagerros YT. The PromMera study – an RCT evaluating the effect of a smartphone application to improve lifestyle after bariatric surgery. Surgery for Obesity and Related Diseases. 2018;14(11). | App not found in app store |
| Ienca R, Giardiello C, Badiuddin F, Rosa M, Pagan A, De Mallorca P, et al. Long-term efficacy of the elipse gastric balloon system: An international multicenter study. Obesity. 2020;28(SUPPL 2). | Name of app not reported |
| Ienca R, Giardiello C, Schiano Di Cola R, Rosa M, Juneja G, Badiuddin F, et al. The evolution of Allurion program for long term weight loss: from virtual monitoring to a virtual care suites. Obesity Facts. 2022;15. | Name of app not reported |
| Jones S, Jones D, DeMaria E, Wynn J, Rosenthal R. Impact of the ASMBS Essentials of Bariatric & Metabolic Surgery App as a Multidisciplinary Online Educational Curriculum. Surgery for Obesity and Related Diseases. 2019;15(10). | Content of app |
| Kabbani J, Kabbani J. Exploring the merits and availability of smartphone applications related to bariatric surgery. British Journal of Surgery. 2021;108(SUPPL 2). | Name of app not reported |
| Kerry G, Gokani S, Ash J, Rasasingam D, Zargaran A, Mittal A, et al. The use of digital education for patients on the bariatric surgery pathway. International Journal of Surgery. 2017;47. | App not found in app store |
| Kim Y, Sysko R, Michaeledes A, Ramos T, Hildebrandt T. Effects of smartphone coaching intervention on dietary intake for bariatric surgery candidates: A pilot randomized controlled trial. CNS Spectrums. 2019;24(1). | Content of app |
| King DC, Purkayastha S, Mobasheri M, Ahmed A, Darzi A. Specifications for an mhealth platform targeted at the bariatric patient pathway. Obesity Surgery. 2014;24(8). | Name of app not reported |
| Klasnja P, Rosenberg DE, Zhou J, Anau J, Gupta A, Arterburn DE. A quality-improvement optimization pilot of BariFit, a mobile health intervention to promote physical activity after bariatric surgery. Translational Behavioral Medicine. 2021;11(2). | No access to app |
| Kulendran M, Lim M, Laws G, Chow A, Nehme J, Darzi A, et al. Surgical smartphone applications across different platforms: their evolution, uses, and users. Surgical Innovation. 2014;21(4). | No relevant app identified |
| Mangieri CW, Johnson RJ, Choi YU, Wood JC, Eisenhower DD. Mobile health applications, do they enhance weight loss efficacy following bariatric surgery. Surgical Endoscopy and Other Interventional Techniques. 2017;31. | Content of app |
| Mangieri CW, Johnson RJ, Sweeney LB, Choi YU, Wood JC. Mobile health applications enhance weight loss efficacy following bariatric surgery. Obesity Research & Clinical Practice. 2019;13(2). | Content of app |
| Masino C, Samuel T, Jimenez MC, Sockalingam S, Jackson T, Quereshy F, et al. Effect of a mobile health patient engagement tool for postoperative care of bariatric surgery patients. Surgery for Obesity and Related Diseases. 2016;12(7). | Name of app not reported |
| McCormack C, Isom K. The effect of smartphone applications and technology on weight loss success and their possible impact on bariatric patients. Surgery for Obesity and Related Diseases. 2015;11(6). | Name of app not reported |
| Messiah SE, Sacher PM, Yudkin J, Ofori A, Qureshi FG, Schneider B, et al. Application and effectiveness of eHealth strategies for metabolic and bariatric surgery patients: A systematic review. Digital Health. 2020;6. | Content of app |
| Morales-Conde S, Alarcón Del Agua I, Aliaga Verdugo A, Román Moyano M, Soria Morillo LM, Escobero Castro M, et al. App for obesity surgery monitoring. Surgical Endoscopy. 2018;32. | Language of app |
| Moura Junior L, Dantas R, Neiva A, Sousa G. Clinical approach to postoperative bariatric emergencies by means of a smartphone application Post-operative complications. Obesity Surgery. 2017;27(1). | Name of app not reported |
| Mundi MS, Lorentz PA, Grothe K, Kellogg TA, Collazo-Clavell ML. Feasibility of Smartphone-Based Education Modules and Ecological Momentary Assessment/Intervention in Pre-bariatric Surgery Patients. Obesity Surgery. 2015;25(10). | Name of app not reported |
| Murphy J, Uttamlal T, Schmidtke KA, Vlaev I, Taylor D, Ahmad M, et al. Tracking physical activity using smart phone apps: assessing the ability of a current app and systematically collecting patient recommendations for future development. BMC Medical Informatics & Decision Making. 2020;20(1). | No relevant app identified |
| Newton R, Clough O, Hawkins W, Pring C, Slater G, Currie A, et al. Establishing the current usage of smartphone technology in bariatric surgery patients. Obesity Surgery. 2019;29(5). | No relevant app identified |
| Nienhuijs S, Van Montfort G, Van Himbeeck F, De Zoete JP, Luyer M, Smulders F. Implementation of ehealth into a bariatric program; 2 years experiences pre-operative management. Obesity Surgery. 2017;27(1). | No access to app |
| Poulsen L, Poulsen S, Larsen BF, Lorenzen M, Roessler KK, Bo Thomsen J, et al. Development of a patient education program for patients undergoing body contouring after massive weight loss. Quality of Life Research. 2018;27. | Name of app not reported |
| Qian T, Yoo H, Klasnja P, Almirall D, Murphy SA. Estimating time-varying causal excursion effect in mobile health with binary outcomes. Biometrika. 2021;108(3). | No access to app |
| Rafols JP, De La Cruz J, Ubach AG, Segarra M. Personalized online monitoring using e-health technology for postoperative follow up after bariatric surgery. A randomized controlled trial. Obesity Surgery. 2014;24(8). | Name of app not reported |
| Rivas H, Leroux E, Morton JM. Mobile health media, a cutting edge technology in managing morbidly obese patients. Obesity Surgery. 2011;21(8). | Name of app not reported |
| Robinson A, Husband A, Slight B, Slight S. The effectiveness of digital health technologies to support surgical patients in changing health behaviours: A systematic review and narrative synthesis. International Journal of Pharmacy Practice. 2020;28. | Name of app not reported |
| Robinson A, Husband AK, Slight RD, Slight SP. Digital technology to support lifestyle and health behaviour changes in surgical patients: systematic review. Bjs Open. 2021;5(2). | Name of app not reported |
| Schonenberger KA, Cossu L, Prendin F, Cappon G, Wu J, Fuchs KL, et al. Digital Solutions to Diagnose and Manage Postbariatric Hypoglycemia. Frontiers in Nutrition. 2022;9. | No relevant app identified |
| Schumacher L, Thomas G, Vithiananthan S, Webster J, Jones D, Bond D. Using novel technology to understand bariatric surgery patients’ physical activity experiences in near real-time through accelerometry-prompted ecological momentary assessment. Surgery for Obesity and Related Diseases. 2019;15(10). | Name of app not reported |
| Shah R, Ioannidis S, Field C, Pisapati G, Caslake Holding F, Patel H, et al. Launching a patient-centred recovery app to assess recovery after weight-loss surgery at a specialist bariatric centre. Anaesthesia. 2018;73. | Name of app not reported |
| Sockalingam S, Ho R, Zhang M. An evaluation of the evidence base of current bariatric surgery and bariatric psychosocial applications. Australian and New Zealand Journal of Psychiatry. 2016;50. | Name of app not reported |
| Sohail S, Shim J, Venkataramanan R, Jaimini U, Berman D, Parikh P, et al. A multisensory approach to monitor bariatric patient's postsurgical behavior and lessen weight recidivism. Surgery for Obesity and Related Diseases. 2017;13(10). | Name of app not reported |
| Spetz K, Hult M, Olbers T, Bonn S, Svedjeholm S, Lagerros YT, et al. A smartphone application to improve adherence to vitamin and mineral supplementation after bariatric surgery. Obesity. 2022;30(10). | App not found in app store |
| Stevens DJ, Jackson JA, Howes N, Morgan J. Obesity surgery smartphone apps: a review. Obesity Surgery. 2014;24(1). | Name of app not reported |
| Sysko R, Michaelides A, Costello K, Herron DM, Hildebrandt T. An Initial Test of the Efficacy of a Digital Health Intervention for Bariatric Surgery Candidates. Obesity Surgery. 2022;32(11). | Name of app not reported |
| Taylor D, Murphy J, Ahmad M, Purkayastha S, Scholtz S, Ramezani R, et al. Quantified-Self for Obesity: Physical Activity Behaviour Sensing to Improve Health Outcomes. Studies in Health Technology & Informatics. 2016;220. | No relevant app identified |
| Tewksbury C, Cassella L, Hesson L, Dumon KR, Williams NN. Healthcare Technology Use among Bariatric Surgery Patients. Surgery for Obesity and Related Diseases. 2018;14(11). | Name of app not reported |
| Tewksbury C, Mulgrew M, Warner-Grimsley S, Countess J, Dumon KR, Williams NN. Qualitative Assessment of Technology Use and Development of a Smartphone App for Bariatric Surgery Patients. Surgery for Obesity and Related Diseases. 2018;14(11). | Name of app not reported |
| Thomas C, Simmons E, Musbahi A, Courtney M, Small P. A contemporary review of smart phone applications in Bariatric & Metabolic Surgery; an underdeveloped UK support service. Obesity Surgery. 2022;32. | Name of app not reported |
| Topart P. Ambulatory remote monitoring of vital signs after fast-Track bariatric surgery: A prospective study evaluating a wireless patch sensor. Surgery for Obesity and Related Diseases. 2017;13(10). | No access to app |
| Versteegden D, Van Himbeeck M, Jf S, De Zoete J, Van Montfort G, Nienhuijs S. A randomized controlled trial for assessing the value of ehealth in bariatric care: 1-year-bepatient trial results. Obesity Surgery. 2019;29(5). | No access to app |
| Versteegden DP, Van Himbeeck M, Nienhuijs SW. Assessing the value of ehealth for bariatric surgery: The bepatient-trial technology and bariatric surgery. Obesity Surgery. 2017;27(1). | No access to app |
| Wright C, Mutsekwa RN, Hamilton K, Campbell KL, Kelly J. Are eHealth interventions for adults who are scheduled for or have undergone bariatric surgery as effective as usual care? A systematic review. Surgery for Obesity & Related Diseases. 2021;17(12). | Name of app not reported |
| Zhang MW, Ho RC, Cassin SE, Hawa R, Sockalingam S. Online and smartphone based cognitive behavioral therapy for bariatric surgery patients: Initial pilot study. Technology & Health Care. 2015;23(6). | Name of app not reported |
| Zhang MW, Ho RC, Hawa R, Sockalingam S. Pilot implementation and user preferences of a Bariatric After-care application. Technology & Health Care. 2015;23(6). | Name of app not reported |
| Zhang MW, Ho RC, Hawa R, Sockalingam S. Analysis of the Information Quality of Bariatric Surgery Smartphone Applications Using the Silberg Scale. Obesity Surgery. 2016;26(1). | No relevant app identified |
| Zhang MW, Ho RC, Sockalingam S. Bariatric surgery smartphone applications: Analysis of information quality. Canadian Journal of Diabetes. 2015;39. | Name of app not reported |
| Zhang MW, Ho RC, Sockalingam S. Online and smartphone based cognitive behavioral therapy for bariatric surgery patients: Initial pilot study. Canadian Journal of Diabetes. 2015;39. | Name of app not reported |
