# Supplementary material for: Content and quality of smartphone applications for bariatric surgery: A review and content analysis
Source: PEC Innov. 2025 Apr 8;6:100391. doi: 10.1016/j.pecinn.2025.100391 (PMC12023771; doi:10.1016/j.pecinn.2025.100391)
Supplement: Supplementary file 4 — Supplementary material 4 [file mmc4.docx]

**Appendix A.4** – Quality assessment based on the MARS tool

|  | **App name** | **Adipositas-Behandlungen** | **Adipositas-Chirurgie** | **Agrundo** |
| --- | --- | --- | --- | --- |
| **Domain** | **Item** | Rating (1-5) | Rating (1-5) | Rating (1-5) |
| **Section B - Functionality** | Performance: How accurately/ fast do the app features (functions) and components (buttons/ menus) work? | 5 | 5 | 4 |
|  | Ease of use: How easy is it to learn how to use the app; how clear are the menu labels/ icons and instructions? | 4 | 3 | 4 |
|  | Navigation: Is moving between screens logical/ accurate/ appropriate/ uninterrupted; are all necessary screen links present? | 4 | 3 | 4 |
|  | Gestural design: Are interactions (taps/ swipes/ pinches/ scrolls) consistent and intuitive across all components/ screens? | 5 | 2 | 4 |
|  | **Functionality mean score (total score: 20)** | **18** | **13** | **16** |
| **Section D - Information** | Accuracy of app description (in app store): Does app contain what is described? | 3 | 1 | 5 |
|  | Goals: Does app have specific, measurable and achievable goals (specified in app store description or within the app itself)? | NA | NA | NA |
|  | Quality of information: Is app content correct, well written, and relevant to the goal/ topic of the app? | 3 | 2 | 4 |
|  | Quantity of information: Is the extent coverage within the scope of the app; and comprehensive but concise? | 2 | 2 | 2 |
|  | Visual information: Is visual explanation of concepts – through charts/ graphs/ images/ videos, etc. – clear, logical, correct? | 2/2* | NA | 2/5* |
|  | Credibility: Does the app come from a legitimate source (specified in app store description or within the app itself)? | 3 | 2 | 3 |
|  | Evidence base: Has the app been trialled/tested; must be verified by evidence (in published scientific literature)? | NA | NA | NA |
|  | **Information mean score (total score: 35)** | **13/13*** | **7** | **16/19*** |

*rating for ilustrations/videos
