# Supplementary material for: Content and quality of smartphone applications for bariatric surgery: A review and content analysis
Source: PEC Innov. 2025 Apr 8;6:100391. doi: 10.1016/j.pecinn.2025.100391 (PMC12023771; doi:10.1016/j.pecinn.2025.100391)
Supplement: Supplementary file 5 — Supplementary material 5 [file mmc5.docx]

**Appendix A.5** – Quality assessment based on APS checklist

| **App name** | | **Adipositas-Behandlungen** | **Adipositas-Chirurgie** | **Agrundo** |
| --- | --- | --- | --- | --- |
| **Domain** | **Item** | Rating (y/n/NA) | Rating (y/n/NA) | Rating (y/n/NA) |
| **1. Purpose and functionality** | 1.1 The specific scope of the app is clearly described, e.g. daily reminders to take medication. | y | n | y |
|  | 1.2. The app lists its own limitations (e.g. disclaimer stating that the app cannot replace a real-life medical consultation). | n | n | n |
|  | 1.3. The last update was provided less than six months ago (as a rule of thumb). | n | n | n |
|  | 1.4. The app runs reliably and does not crash or restart unprompted. | y | y | y |
| **2. Quality and Evaluation** | 2.1. The app does not provide a definitive diagnosis and corresponding treatment recommendations. | y | y | y |
|  | 2.2. The app does support existing treatment plans (e.g. by recording biometrics). | n | n | y |
| **3. Ratings by other users** | The app has frequently been rated positively by a many different users. | NA | NA | NA |
| **4. Quality certificates and certification marks** | 4.1. The app has been certified and awarded a trustworthy certification mark. | n | n | n |
|  | 4.2. The evaluation criteria associated with the certification mark are clear and understandable. | NA | NA | NA |
| **5. Data privacy notice** | 5.1. The app provides a data privacy notice, which… | n | n | y |
|  | 5.1.1 is easy to find and to view (e.g. directly within the application, on its websites or in app stores), | NA | NA | y |
|  | 5.1.2 provides information about the type, extent and purpose of data collection as well as the particulars of how data are processed and whether they are passed on to third parties, | NA | NA | y |
|  | 5.1.3 specifies where (in which country) and how (encrypted/anonymized) your data are stored, | NA | NA | n |
|  | 5.1.4 specifies whether and how (via email or post etc.) you can withdraw your consent for data storage. | NA | NA | n |
| **6. Access to functionality and data** | 6.1 The app only requests data that are important to its functionality. | y | y | y |
|  | 6.2. Requests for access to functions of the mobile device (e.g. access to location via GPS or to the calendar) are only requested in order to facilitate app usage. | NA | NA | NA |
|  | 6.3. The app specifies that it is possible for collected and stored data to be deleted. Given that it is rarely possible to delete data irretrievably, you should be careful in deciding which data to share. | NA | NA | n |
| **7. Imprint** | 7.1. The name and address of the provider. Legal entities (e.g. Inc., PLC, Ltd., Co.) in particular must state their legal form and authorized representatives. | n | n | n |
|  | 7.2. Details for direct and immediate contact (telephone or fax number, email address). | y | y | y |
| **8. Funding and financial background** | 8.1. The financial background of an app is apparent if, for example, - users pay for the app and it finances itself, - the costs for the app are reimbursed by health insurance providers (this is very rare), - sponsors and/or public funding finance the app. | n | n | n |
|  | 8.2. The app is neutral, meaning that its contents are not influenced by commercial interests, if for example, • the app does not advertise a specific product, • the app is provided by a public or charitable organization. | y | y | y |
| **Overall quality assessment** | **Does the app meet the quality criteria of the APS *checklist for the use of health apps*?** | **n** | **n** | **n** |
